# G9a Inhibition Induces Autophagic Cell Death *via* AMPK/mTOR Pathway in Bladder Transitional Cell Carcinoma

**DOI:** 10.1371/journal.pone.0138390

**Published:** 2015-09-23

**Authors:** Feng Li, Jin Zeng, Yang Gao, Zhenfeng Guan, Zhenkun Ma, Qi Shi, Chong Du, Jing Jia, Shan Xu, Xinyang Wang, Luke Chang, Dalin He, Peng Guo

**Affiliations:** Department of Urology, the First Affiliated Hospital of Xi’an Jiaotong University, Xi’an, Shaanxi, China; Peking University Health Science Center, CHINA

## Abstract

G9a has been reported to highly express in bladder transitional cell carcinoma (TCC) and G9a inhibition significantly attenuates cell proliferation, but the underlying mechanism is not fully understood. The present study aimed at examining the potential role of autophagy in the anti-proliferation effect of G9a inhibition on TCC T24 and UMUC-3 cell lines *in vitro*. We found that both pharmaceutical and genetical G9a inhibition significantly attenuated cell proliferation by MTT assay, Brdu incorporation assay and colony formation assay. G9a inhibition induced autophagy like morphology as determined by transmission electron microscope and LC-3 fluorescence assay. In addition, autophagy flux was induced by G9a inhibition in TCC cells, as determined by p62 turnover assay and LC-3 turnover assay. The autophagy induced positively contributed to the inhibition of cell proliferation because the growth attenuation capacity of G9a inhibition was reversed by autophagy inhibitors 3-MA. Mechanically, AMPK/mTOR pathway was identified to be involved in the regulation of G9a inhibition induced autophagy. Intensively activating mTOR by Rheb overexpression attenuated autophagy and autophagic cell death induced by G9a inhibition. In addition, pre-inhibiting AMPK by Compound C attenuated autophagy together with the anti-proliferation effect induced by G9a inhibition while pre-activating AMPK by AICAR enhanced them. In conclusion, our results indicate that G9a inhibition induces autophagy through activating AMPK/mTOR pathway and the autophagy induced positively contributes to the inhibition of cell proliferation in TCC cells. These findings shed some light on the functional role of G9a in cell metabolism and suggest that G9a might be a therapeutic target in bladder TCC in the future.

## Introduction

In neoplasms deriving from bladder, 95% was transitional cell carcinoma (TCC), which accounted for nearly 4.5% of adult malignancy in 2014[1]. Non-muscle-invasive bladder cancer (NMIBC), which represents approximately 75% of the patients, and muscle-invasive disease (MIBC) which takes up the remaining part are two subgroups of TCC[2,3]. In NMIBC, 60% patients are expected to recurrence and 20% of them will develop a MIBC. The overall survival rate of NMIBC patients is around 88–98% after 5 five years. However, for those with muscle-invasive disease, half of them are expected to die within 5 years[4]. Transurethral resection (TUR) is essential to remove all visible lesions in NMIBC while radical cystectomy (RC) with lymphadenectomy is needed for MIBC. After surgery, Bladder irrigation using chemotherapeutic drugs like pharmorubicin has been recommended to lower recurrence and mortality[5]. Since the occurrence and recurrence rates of TCC are high, it is urgent to search for genetic targets to cure bladder cancer thoroughly[6].

G9a/EHMT2, a histone methyltransferase which catalysts the 9^th^ lysine of histone 3, has been demonstrated to be an oncogene in bladder cancer and it may play pivotal role in the initiation of bladder neoplasms[7,8]. G9a has also been presented to highly express in several cancers like head and neck squamous cell carcinoma and breast cancer. Increasing expression of G9a is positively correlated to cell proliferation. G9a inhibition both pharmaceutically and genetically significantly inhibits cell proliferation by inducing cell cycle arrest, triggering apoptosis or inducing autophagic cell death[9–14].

Autophagy, an evolutionarily conserved self-eating process degrading cytoplasmic proteins and organelles is essential for cellular homeostasis[15,16]. Recent evidence suggests that autophagy may act as “double-edged swords” towards cell growth. It can promote cell survive *via* degrading unnecessary molecules or organelles to supply materials that is needed for cell metabolism. On the other hand, autophagy may interplay with apoptosis or cell cycle arrest or directly trigger autophagic cell death, which subsequently leads to inhibition of cancers[17–19]. G9a inhibition has been demonstrated to trigger apoptosis in TCC[20], while whether G9a inhibition could induce autophagy and what is the role of autophagy induced to cell proliferation in TCC remains to be elucidated. In the present study, we identified whether inhibition of G9a could induce autophagy, and the role of autophagy towards cell proliferation in TCC T24 and UMUC-3 cell lines, and further investigated whether the autophagy depends on AMPK/mTOR pathway.

## Materials and Methods

### Cell culture

TCC cell lines T24 and UMUC-3 were purchased from American Type Culture Collection (ATCC) and cultured in Dulbecco's Modified Eagle Medium (DMEM). Culture medium was supplemented with 10% fetal bovine serum and 100 U/ml penicillin and 0.1 mg/ml streptomycin (Gibico). Cells were incubated in a humidified atmosphere contains 5% CO_2_ at 37°C and observed by inverted microscope (×100 and ×200, Olympus).

### Reagents, antibodies and plasmids

BIX-01294 (S8006) was purchased from Selleckchem, 3-methyadenine (3-MA, 189490) and Bafilomycin A1 (BAFA1, 196000) were purchased from EMD Millipore. Chloroquine (CQ, C6628) was from Sigma. Compound C (ab120843) and AICAR (ab120358) were purchased from Abcam. Lipofectamine 2000 reagent was purchased from Invitrogen. RIPA buffer was purchased from Cell Signaling Technology (CST), protease inhibitor and phosphatase inhibitor were from Roche. BCA qualification system was purchased from Pierce. Primary antibodies against LC-3 I/II, ATG3, ATG5, ATG7, p-Raptor (Ser792), Raptor, mTOR, p-mTOR(Ser2448), p-ACC (Ser79), p-AMPK α (Thr172), AMPK α, p-S6K (Thr389), p-4E-BP1 (Thr37/46), histone 3, Rheb, β-actin and peroxidase-conjugated secondary antibodies were purchased from Cell Signaling Technology (CST), p62 was from Novus, H3k9me2 was from Abcam. PVDF membrane was purchased from Bio-rad. The shG9a #1 and shG9a #2 plasmids and a scrambled RNA which used as shcontrol were purchased from GenePharma, the target sequence was shown in S1 Table. GV230-Rheb plasmid was constructed by GeneChem (Gene accession NM_005614), and empty GV230 vector was used as the control. Neromycin was used to screen steady cloning The mRFP-EGFP-LC-3 reporter plasmid (ptfLC-3) was a gift from Tamotsu Yoshimori (Addgene plasmid # 21074)[21].

### Cell Viability Test

Cell viability was assessed by using 3-(4, 5-dimethylthiazol-2-yl)-2, 5-diphenyltetrazolium bromide (MTT) assay. 3000 cells in 100 μl of medium per well were seeded in 96-well plates. Cells were treated as indicated and cultured for the indicated time, and then incubated with 0.5 mg/ml of MTT at 37°C for 4h. Medium was replaced by 150 μl DMSO per well to dissolve the precipitates. Colorimetric analysis using a 96-well micro-plate reader (Bio Tek) was performed at wavelength 490 nm.

### Brdu incorporation assay

Cells were seeded to 24 well plate and treated with BIX-01294 1.5 μM for 48 h or transfected with shG9a #1 plasmid for 96 h. Brdu (20 μM/ml) was added to the medium 4 h before harvesting. Then cells were fixed in paraformaldehyde for 20min and then 0.1% Triton X-100 for 10min. Incubated in HCl (2 M) for 10min at room temperature and then HCl (1 M) for 10min on ice. Neutralized by incubating the samples in borate buffer (0.1 M) for 10 min at room temperature. Then samples were incubated in 1% BSA to block the non-specific antigen for 1h at room temperature and the anti-Brdu diluted in 1% BSA was added overnight. Washed by PBS for 3 times and incubated with TRITC labeled goat anti-mouse for 1h at room temperature. Washed by PBS for 3 times and stained the nuclear by DAPI (1μg/ml) for 1min. Washed by PBS again, observed and captured by fluorescence microscope (×100, Olympus).

### Colony formation assay

Cells were seeded in 6-well plate (1,000 cells/well) in 2 ml culture medium overnight. In drug treatment group, the medium was changed with fresh medium containing BIX-01294 (0.75 and 1.5 μM) or vehicle (DMSO) every other 2 days. In gene interference group, cells were transfected with shG9a #1, shG9a #2 and shcontrol (a scrambled RNA), culture medium were changed every other day. Then both groups continued to keep cells growing for two weeks. Colonies were fixed with 4% paraformaldehyde and stained by crystal violet for 10 min respectively at room temperature. Colonies consisted of more than 50 cells were counted.

### Detection of Apoptosis by Flow-cytometric Analysis

Cells were treated with BIX-01294 1.5μM and DMSO for 48 h or transfected with shG9a #1, shG9a #2 or a scrambled RNA for 96 h. Then cells were collected and subjected to Annexin V and propidium iodide (PI) staining using an Annexin V-FITC Apoptosis Detection Kit, following the protocols provided by the manufacturer. Apoptotic cells were then analyzed by flow-cytometry (BD FACSCalibur).

### Cell cycle detection assay

Cells were treated as the treatment in apoptosis detection assay and then washed twice with PBS, fixed with 70% ethanol overnight at -20°C, and re-suspended with PI solution (0.05 mg/ml) containing RNase and incubated at room temperature in the dark for 30 min. DNA content was then analyzed by using the flow-cytometry (BD FACSCalibur).

### Tandem fluorescence microscopy assay

Cells were seeded onto coverslip and transient transfected with tandem fluorescent ptfLC-3 expressing plasmid with Lipofectamine 2000 reagent. After 24 h transfection, cells were treated with BIX-01294 1 μM and DMSO for additional 24 h or transfected with shG9a #1 and a scrambled RNA for 72 h. Cells were then fixed by 4% paraformaldehyde. The localization of LC-3 puncta was observed by fluorescence microscopy (×200, Olympus).

### Transmission electron microscopy assay

Cells were treated with BIX-01294 1 μM and DMSO for additional 24 h or transfected by shG9a #1 and a scrambled RNA for 72 h and then fixed in Karnovsky's fixative (2% paraformaldehyde and 5% glutaraldehyde in 0.1 M cacodylate, pH 7.4) followed by osmium tetroxide. Samples were then dehydrated in ethanol, infiltrated and embedded with TAAB Low Viscosity Resin (TLV) mixture at 60°C for 24 h and sectioned to 80nm in thickness on 300 mesh copper slot grids. Analysis was performed by transmission electron microscopy (×8,000 and ×40,000, JEOL, JEM-1400).

### Western blot analysis

Cells were treated with designated treatments and then washed with ice-cold phosphate-buffered saline (PBS) and then solubilized in RIPA buffer, containing protease inhibitor and phosphatase inhibitor. Lysates were quantified by BCA quantification system. Then protein was loaded to 12% SDS-PAGE and transferred to PVDF membrane. Membranes were immune-blotted with indicated primary antibodies (dilution: 1:1,000) overnight at 4°C followed by peroxidase-conjugated secondary antibody (dilution: 1:3,000) for 1 hour at room temperature. The bands were visualized by ECL system (Bio-rad).

### Quantitative real-time polymerase chain reaction (RT-PCR) analysis

The protocol used to extract sample RNA and the information of samples were described elsewhere[22], All the experiments involved in human tissue are approved by the Ethics committee of Xi’an Jiaotong University. Total RNA from cells was extracted using fasten 2000 RNA extract kit following the manufacturer’s protocol. Reverse transcription was performed with 2 μg RNA using Takara reverse kit. SYBR green reaction mix (Takara) was used to perform RT-PCR following the manufacturer’s instructions. Primer sequences used were shown in S2 Table. β2mircoglobulin (β2MG), a mostly used internal control during autophagy, was used as the control.

### Statistical analysis

All experiments were performed at least three times. The results were presented as the mean ± standard deviation (SD). One-way ANOVA was performed to analyze differences between experimental groups. Image analyzer Image J (NIH) was used to quantify LC-3 puncta numbers. A *P* value of <0.05 was considered statistically significant in all cases.

## Results

### G9a expression is higher in TCC

**Fig 1:**
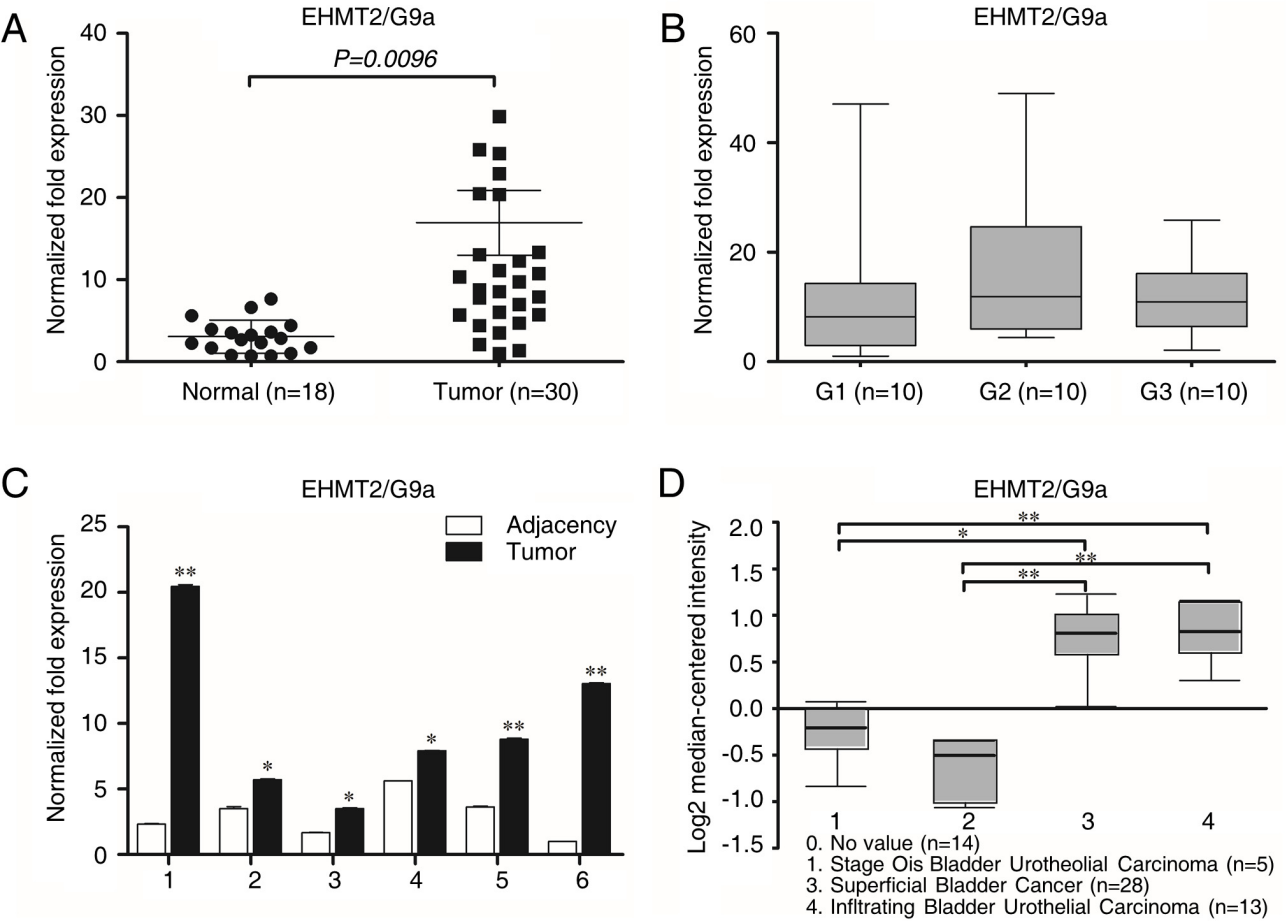
G9a’s expression is higher in TCC. The mRNA expression of G9a between TCC samples and normal bladder tissues **(A)**, among the grades of TCC samples **(B)** and between paired solid tumor and its adjacency **(C)**. **(D)** The expression of G9a in bladder normal tissues and cancers from www.oncomine.org. *P < 0.05; **P<0.01.

Since G9a has been demonstrated to express high in various cancers including TCC, we firstly checked its expression in the specimens collected by our department. As shown in Fig 1A and 1B, the expression of G9a was higher in tumors comparing to normal bladder tissues and there was no difference among tumor stages. In the specimens tested, six were paired as one was from the solid tumor and the other was from adjacent normal tissues. It was obvious that the expression of G9a was higher in tumors than its normal adjacency (Fig 1C). We also searched database from www.oncomine.org and got the similar results (Fig 1D)[23]. These results confirm the oncogene role of G9a in TCC as stated by another group[7].

### Inhibition of G9a attenuates cell proliferation in TCC T24 and UMUC-3 cell lines

**Fig 2:**
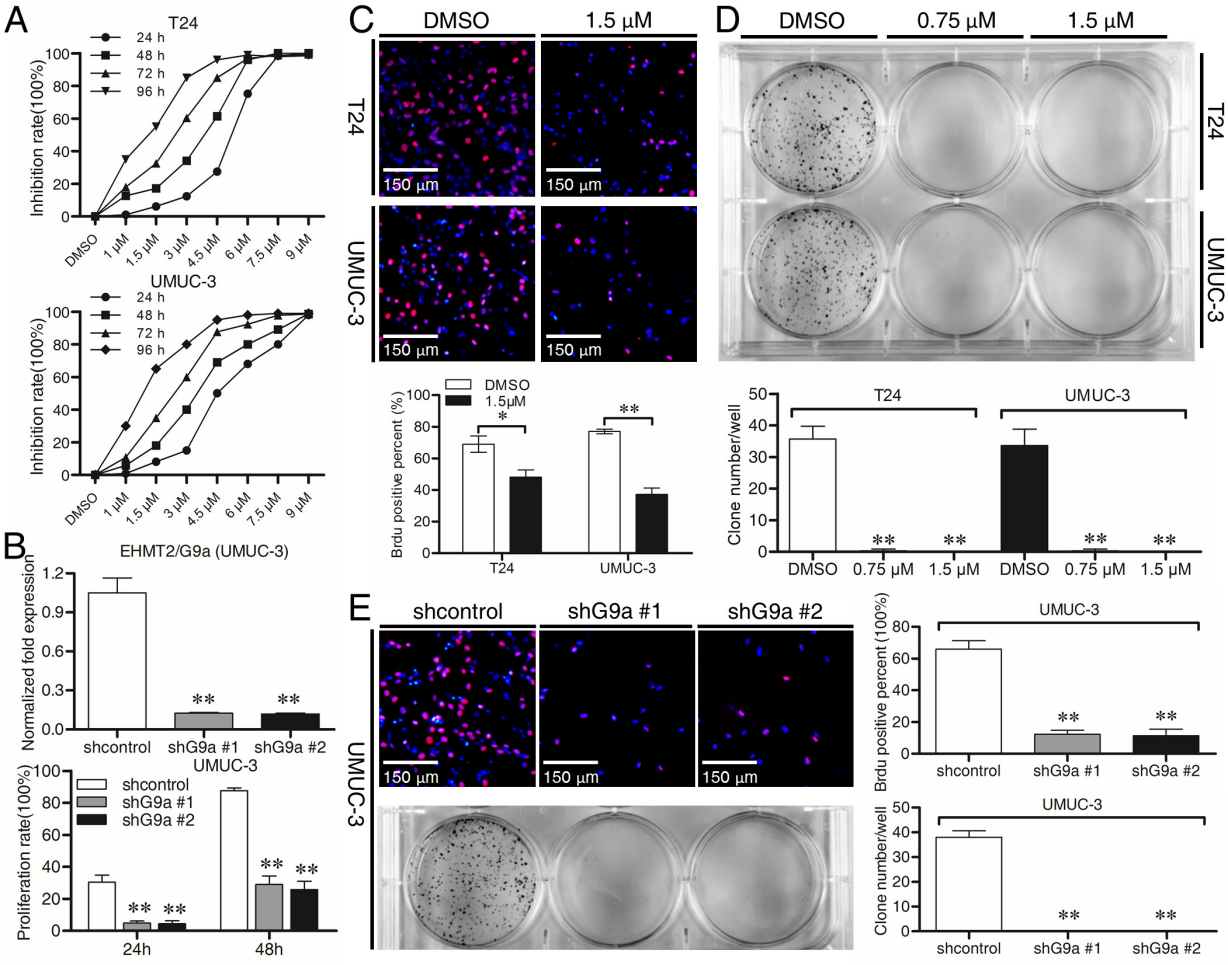
G9a inhibition attenuates cell proliferation in TCC T24 and UMUC-3 cell lines. **(A** and **B)** Cells were treated with G9a inhibitor BIX-01294 at various doses for three time points (24 h, 48 h and 72h). UMUC-3 cells were transfected with shG9a for 48 h, then cells were seeded and cultured for additional two time points (24 h and 48 h), cell viability was determined by MTT assay. **(C)** Cells were treated by BIX-01294 1.5 μM for 48 h and then Brdu incorporation assay was taken out (×100). **(D)** In colony formation assay, cells were treated with Bix-01294 (0.75μM and 1.5μM) for two weeks and colonies were stained by crystal violet and statistically analyzed. **(E)** Brdu incorporation assay and colony formation assay were performed on shRNA transfected UMUC-3 cells. *P < 0.05; **P<0.01.

BIX-01294, a classic and most used G9a inhibitor which inhibits G9a’s catalytic function efficiently, was used to inhibit G9a pharmaceutically;Moreover, two independent shRNA, shG9a #1 and shG9a #2 were used to genetically inhibit G9a while a scrambled RNA was used as the shcontrol. As shown in Fig 2A and 2B, G9a inhibition suppressed proliferation of T24 and UMUC-3 cells as detected by MTT assay. The anti-proliferation effect of BIX-01294 was quite time-dependent as 1.5 μM dose which was lower than the IC50 (about 4.5μM) in 48 h exerted more efficient proliferation-inhibition capacity in 96 h or latter. In addition, Brdu incorporation assay was introduced to evaluate the proliferation of attached cells. When G9a was inhibited, cell growth was attenuated as the percent of Brdu positive cells was far less than control (Fig 2C and 2E). To evaluate the anti-proliferation capacity of G9a inhibition in long-term run, colony formation assay was performed. As shown in Fig 2D and 2E, G9a inhibition could significantly inhibit the formation of colonies after two weeks’ cell culturing. Importantly, the doses of BIX-01294 used in above experiments do not affect normal urologic cells viability much (S1 Fig). These results indicate G9a a therapeutic target in TCC cells.

### Cell proliferation attenuation by G9a inhibition is not via triggering apoptosis or inducing cell cycle arrest

**Fig 3:**
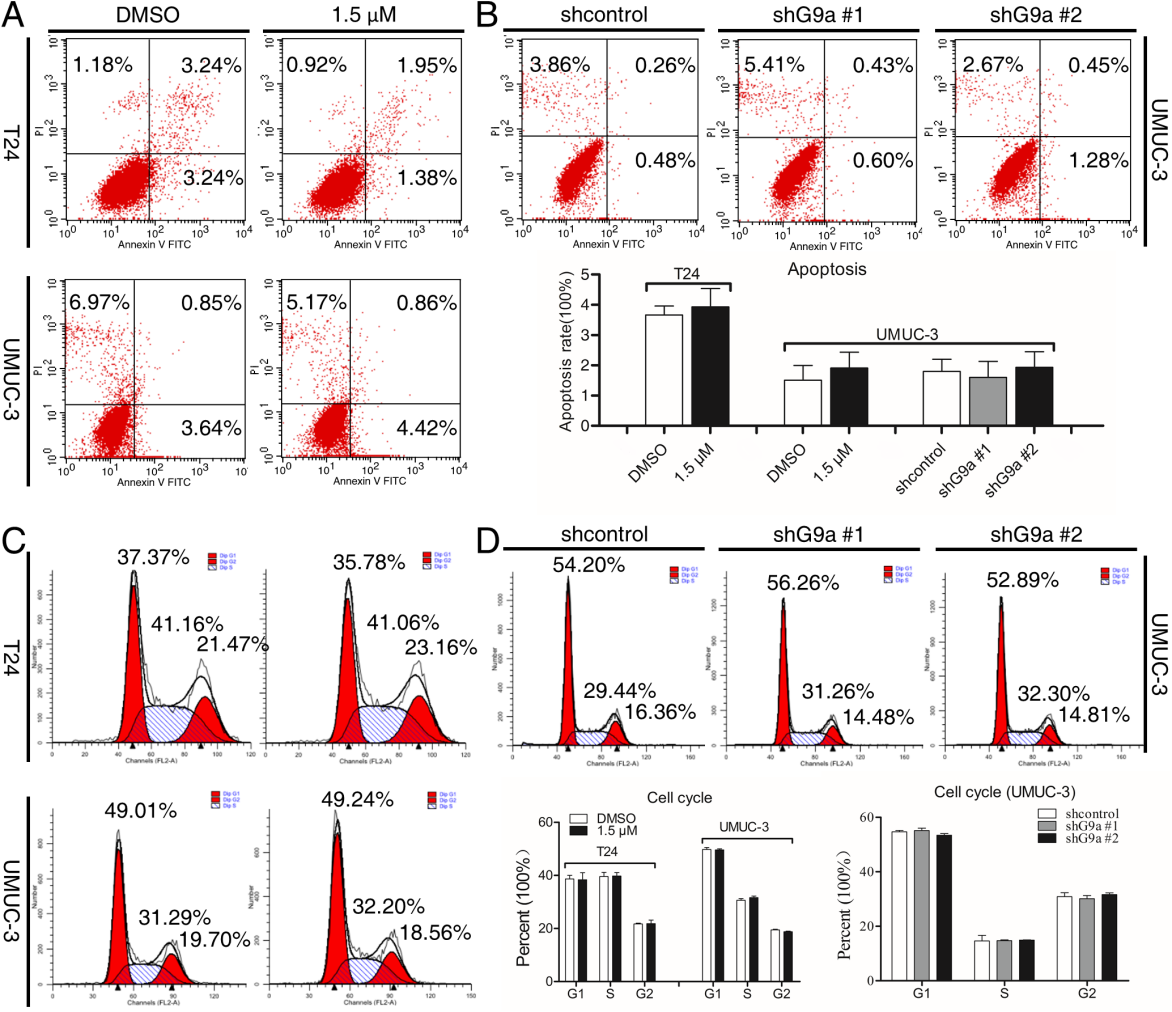
Cell proliferation attenuation by G9a inhibition is not *via* triggering apoptosis or inducing cell cycle arrest. Flow-cytometry was used to determine apoptosis **(A** and **B)** and the distribution of cell cycle phases **(C** and **D)** of cells treated with G9a inhibitor BIX-01294 1.5 μM for 48 h or transfected with shG9a for 96 h. The ratio of apoptotic cells and the percent of each cell phase were counted and analyzed. There was no statistic difference between each group.

BIX-01294 has been demonstrated to trigger apoptosis in TCC, however the doses used (5 and 10 μM) are much higher than the initial proliferation-inhibitory doses of BIX-01294 and whether these doses of BIX-01294 trigger apoptosis or induce cell cycle arrest remains unclear. Flow-cytometry was then performed to check the status of apoptosis and cell cycle of TCC cells when G9a was inhibited. Unexpectedly, G9a inhibition did not elevate the ratio of apoptotic cells (Fig 3A and 3B) and had no effect on the distribution of cell phases (Fig 3C and 3D). These data suggest that apoptosis might be triggered at high doses of BIX-01294, but it is not dominant in the initial doses as well as cell cycle arrest. Other proliferation inhibitory programs might be involved when G9a is inhibited.

### G9a inhibition induces autophagic vacuoles in T24 and UMUC-3 cells

**Fig 4:**
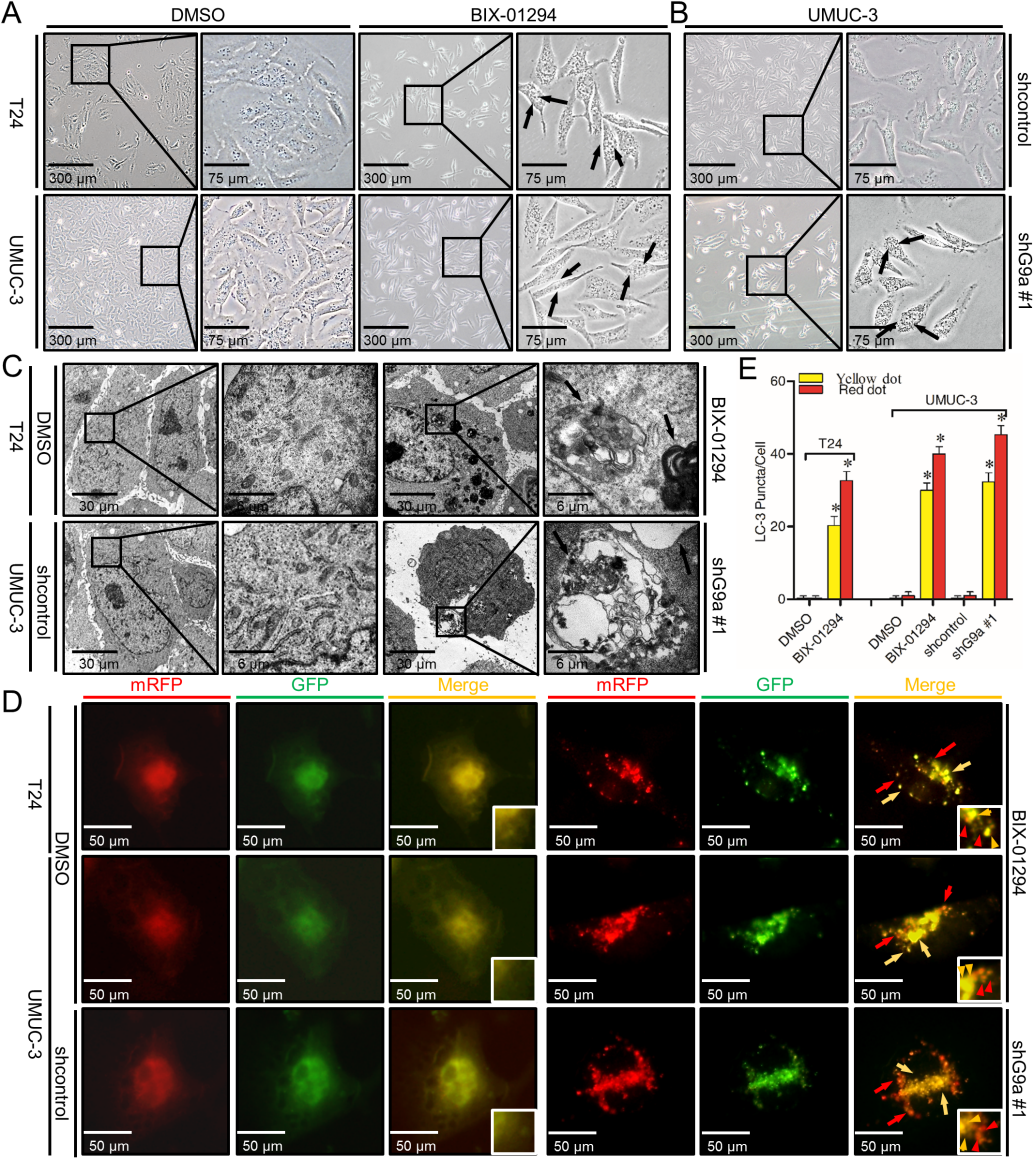
G9a inhibition induces autophagic vacuoles in T24 and UMUC-3. **(A** and **B)** Morphological changes of T24 and UMUC-3 cells after treatment of 1.5 μM BIX-01294 for 24 h or UMUC-3 cells transfected by shG9a #1 for 72 h by inverted phase contrast microscopy (×100 and ×200). Arrows point to cytoplasmic vacuole accumulation. **(C)** Representative electron micrographs of T24 cells treated with 1 μM BIX-01294 for 24 h and 72 h transfected UMUC-3 cells (×8000 and ×40,000). Arrows point to distinct autophagic structures. **(D)** Examples of cells transiently transfected with ptfLC-3 plasmid and treated with 1 μM BIX-01294 for 24 h or transfected with shG9a #1 for 72h under fluorescence microscope (×200). Yellow arrows point to autophagosomes while red arrows point to autolysosomes. **(E)** Quantification of the number of autophagosomes (yellow LC-3 puncta) and autolysosomes (red LC-3 puncta) per cell. Rectangle indicates the magnifying picture right to it in the same treatment group. *P < 0.01.

We noticed that TCC cells in which G9a was inhibited showed morphological features of cytoplasmic vacuole accumulation which resembled autophagy under an inverted microscope (Fig 4A and 4B). These disorganized vacuoles were confirmed to be autophagy related by transmission electron microscopy (TEM) as the ultrastructure of the cells was observed. G9a inhibition significantly increased autophagic double-membrane compartments containing lamellar structures (Fig 4C).

The conversion from LC-3 I to LC-3 II and then trans-localized to autophagic vacuoles during autophagy induction is a hallmark of mammalian autophagy. We next transiently transfected a pH-sensitive LC-3 construct consisting of a tandem fusion of the acid-insensitive mRFP and the acid-sensitive EGFP into TCC cells to address the effects of G9a inhibition on the formation of autophagosome and its matured form autolysosome. The mRFP-EGFP-LC-3 reporter plasmid (ptfLC-3) emits yellow (green merged with red) fluorescence in non-acidic structures (including autophagosome) but red fluorescence only in autolysosomes due to the quenching of GFP at low pH. As shown in Fig 4D and 4E, a pronounced increase in both yellow and red puncta was observed in TCC cells when G9a was inhibited. These data suggest that G9a inhibition enhances autophagosome formation and maturation in TCC cells.

### G9a inhibition induces autophagy flux and autophagic cell death in T24 and UMUC-3 cells

**Fig 5:**
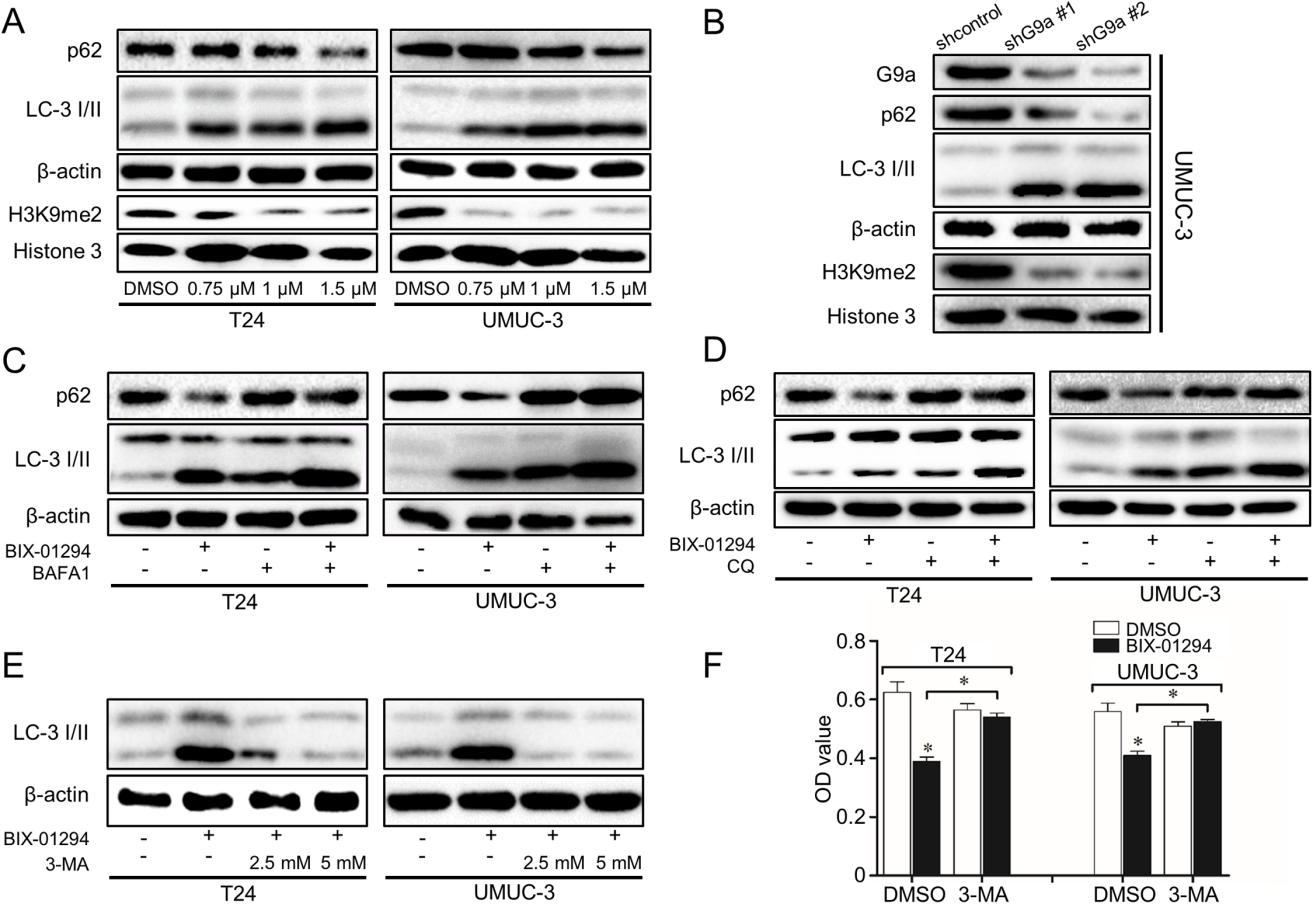
G9a inhibition induces autophagy flux and autophagic cell death in T24 and UMUC-3. **(A** and **B)** Cells were treated with BIX-01294 (0.75, 1 and 1.5μM) or vehicle (DMSO) for 24 h or UMUC-3 transfected with shG9a or a scrambled RNA for 72 h, autophagy markers p62 and LC-3 I/II were checked by Western-Blot. Cells were treated with BIX-01294 1 μM with or without 10 nM BAFA1 **(C)** or 50 μM CQ **(D)**. The levels of LC-3 I/II and p62 were examined. **(E)** The autophagy inhibition effect of 3-MA (2.5 and 5 mM) with the presence of 1.5 μM BIX-01294. **(F)** Cells were pre-treated with 3-MA 5 mM for 4 h and then BIX-01294 1.5μM was added for additional 48 h. Cells viability was assessed by MTT assay. *P<0.01.

Since autophagic morphological feature changes have been observed by G9a inhibition in TCC cells, we further examined the expression of autophagy related markers using western-blot analysis. As shown in Fig 5A, comparing to the control, G9a inhibition resulted in a significant increase of LC-3 II accumulation which was in parallel with the decrease of H3K9me2, a marker which indicates the activity of G9a. More importantly, BIX-01294 could increase the level of LC-3 II at very low doses and at earlier times (S1 Fig).

SQSTM1/p62, a classical macroautophagy substrate which represents autophagic clearance, was then investigated. G9a inhibition resulted in a significant decrease of p62 level (Fig 5A and 5B), suggesting the induction of autophagic clearance.

To further confirm the induction of autophagic flux by G9a inhibition, LC-3 turnover assay was introduced. Bafilomycin A1 (BAFA1), an autophagy inhibitor, can affect the acidification of lysosome to make it nonfunctional to digest LC-3 II and other contents. As expected, BIX-01294 and BAFA1 both elevated LC-3 II levels and LC-3 yellow puncta. However, when BIX-01294 and BAFA1 were combined, the number of yellow puncta and the level of LC-3 II were significantly higher. In addition, the decrease of p62 by BIX-01294 was reversed by BAFA1 (Fig 5C and S2 Fig). The autophagic flux induced by BIX-01294 was further confirmed by another classic lysosome inhibitor chloroquine (CQ) (Fig 5D). These data suggest that the increase in LC-3 II levels by G9a inhibition is a result of autophagy induction rather than decreased recycling of LC-3 II.

Autophagic cell death is a term firstly introduced in 1980s. It is used to describe dying cells that contain numerous autolysosomes and autophagosomes and lack the characteristics of other types of cell death, and the blockade of autophagy (by pharmacological inhibitors like 3-MA, or by genetically interference of autophagy genes) can prevent or delay the death of cells[24]. Since G9a inhibition induces autophagy and attenuates cell proliferation, whether they have some crosstalk remains unclear. Interestingly, when TCC cells were pre-treated with autophagy inhibitor 3-MA, a PI3K inhibitor, the autophagy induced was inhibited (Fig 5E) and the cell proliferation inhibition capacity of BIX-01294 was reversed in MTT assay (Fig 5F). These results suggest that G9a inhibition induces autophagy and the autophagy induced positively contributes to inhibit cell proliferation.

### G9a inhibition induces autophagy and autophagic cell death through AMPK/mTOR pathway

To further explore the underlying mechanism of autophagy induced by G9a inhibition, we examined the expression of autophagy related genes[25]. However, we failed to observe significant changes after BIX-01294 treatment at both mRNA and protein levels (S3 Fig).

**Fig 6:**
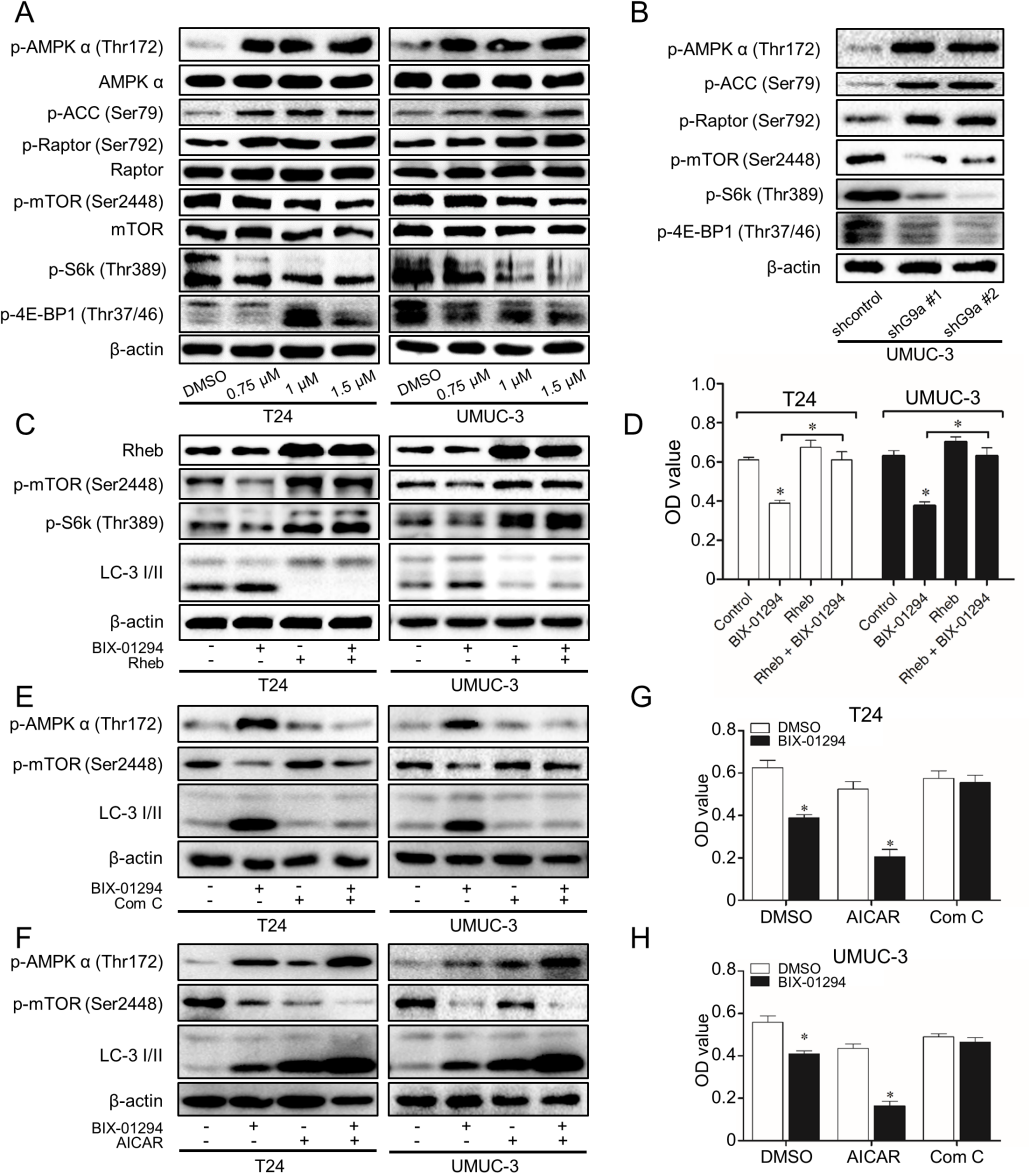
G9a inhibition induces autophagy and autophagic cell death through AMPK/mTOR pathway. **(A** and **B)** Cells were treated with BIX-01294 (0.75 μM, 1 μM and 1.5μM) or vehicle (DMSO) for 24 h, UMUC-3 was transfected with shG9a or a scrambled RNA for 72 h, the protein levels of H3K9me2, p-AMPKα, p-ACC, p-Raptor, p-mTOR, p-S6K and p-4E-BP1 were checked. Cells were stable transfected with GV230-Rheb or empty GV230 vector and then treated with BIX-01294 (1.5 μM) for 24 h to exam the levels of p-mTOR, p-S6K and LC-3 I/II **(C)** or 48 h to assess cell viability by MTT assay **(D)**. Cells were pre-incubated with AMPK inhibitor compound C (5 μM) **(E)** or activator AICAR (1 mM) **(F)** for 4 h and then treated with BIX-01294 (1.5 μM) for 24 h. The levels of p-AMPKα, p-mTOR and LC-3 I/II was examined. T24 **(G)** and UMUC-3 **(H)** were pre-treated with compound C (5 μM) or AICAR (1 mM) and then treated with BIX-01294 (1.5 μM) for 48 h, Cells viability was assessed by MTT assay. *P<0.01.

mTOR complex 1 (mTORC1) signaling is critical for autophagy induction. Activated mTORC1 suppressing autophagy, while repression of mTORC1 inducing autophagy[16]. To examine the role of mTORC1 signaling in G9a inhibition-induced autophagy, we checked the phosphorylation levels of mTOR and its two targets 4E-BP1 and S6K. Our results showed that G9a inhibition inhibited the phosphorylation levels of mTOR at Ser-2448, 4E-BP1 at Thr-37/46 and S6K at Thr-389 (Fig 6A and 6B). To confirm the regulation role of mTOR in G9a inhibition induced autophagy, we next overexpressed Rheb, which intensively activates mTOR in TCC T24 and UMUC-3[26]. As shown in Fig 6C and 6D, Rheb overexpression reversed the autophagy induction capacity of BIX-01294 together with its proliferation inhibition ability, indicating that the autophagy induced by G9a inhibition is mTOR-dependent.

AMP activated protein kinase (AMPK), a key energy sensor, which has been implied to affect mTORC1 to regulate autophagy, was then evaluated when G9a was inhibited. Our results showed that G9a inhibition significantly increased the level of phospho-AMPK (Thr172) and its target phospho-ACC (Ser79), suggesting the activation of AMPK signaling (Fig 6A and 6B). The regulatory associated protein of mTOR (Raptor) can be phosphorylated by AMPK at Ser722/Ser792 and is a negative regulator of mTOR. Once phosphorylated, it departs from mTOR complex to decrease mTOR signaling[27]. G9a increased phosphorylation of Raptor at Ser792, which was in consistent with the raise of phospho-AMPK (Thr172) and the decrease of phospho-mTOR (Ser-2448) (Fig 6A and 6B).

To confirm whether the inhibition of mTOR was due to the increase of AMPK phosphorylation during autophagy, we pre-inhibited AMPK by compound C before BIX-01294 was added. As expected, the effects of BIX-01294 on phospho-mTOR, LC-3 II and the inhibition of proliferation could be reversed by compound C (Fig 6E, 6G and 6H). On the contrary, when AMPK was pre-activated by AICAR, the level of phospho-mTOR, LC-3 II and proliferation attenuation was enhanced (Fig 6F, 6G and 6H).

Collectively, these findings suggest the induction of autophagy and autophagic cell death by G9a inhibition in TCC cells is mTOR-dependent and mediated by AMPK activation.

## Discussion

G9a has been demonstrated to express high in TCC and is correlated with cells proliferation, G9a inhibition results in a significantly proliferation attenuation, however the underlying mechanism is not fully understood. In the present study, we first documented that G9a inhibition induced early autophagy that subsequently contributed to the inhibition of cell proliferation in TCC T24 and UMUC-3 cells *in vitro*, involving activation of AMPK/mTOR pathway.

Autophagy induced by anticancer reagents or gene silence has been widely studied in various cancer cell models[28]. Abundant evidence shows that autophagy may play dual roles in carcinogenesis and anticancer therapies which depends on the contents degraded. Autophagy degrades unfolded or aggregated proteins and organelles to maintain intracellular metabolic homeostasis and thus facilitates the cancer cells resistance to chemotherapy and radiation treatment[29]. On the other hand, it may degrade BCL2, an anti-apoptotic protein, to trigger apoptosis[30]. G9a inhibition, no matter genetically or pharmaceutically, has been reported to induce autophagic cell death in various cancers[31,32]. In our study, we first document that G9a inhibition induces autophagy in TCC T24 and UMUC-3 cells, as determined by electron microscopy, LC-3 turnover assay, p62 turnover assay and tandem fluorescence microscopy.

Several critical molecules and pathways have been demonstrated to regulate autophagy progress. The best characterized is AMPK/mTOR pathway and it attracts great attention of researchers[33,34]. Ulk1/2-Atg13-FIP200 complex is considered to be the initial step of autophagosome biogenesis and mTORC1 crosstalk with it via direct interaction between Raptor and Ulk1/2. Active mTOR1 phosphorylates Atg13 and Ulk1/2, thereby suppressing Ulk1/2 kinase activity[35]. The network of AMPK inducing autophagy is more complicate. AMPK mediates the activity of mTORC1 *via* phosphorylating Raptor and TSC2, two negative regulator of mTORC1, to induce autophagy[36,37]. Meanwhile, AMPK could directly interact with Ulk1 and positively regulate its activity through AMPK-dependent phosphorylation, further enlarges the range of possibilities for AMPK to induce autophagy[38]. As feedback, Ulk1 in turn directly interferes with mTORC1 signaling *via* Ulk1-dependent phosphorylation of raptor, which either results in direct inhibition of mTORC1 kinase activity or interferes with raptor-substrate interaction, finally leads to reduced phosphorylation of mTORC1 and downstream targets[39]. Activated Ulk1 also phosphorylates AMPK and inhibits its activation, providing another potential negative-feedback loop on autophagy induction[40,41]. Our further mechanistic studies revealed that the autophagy induction by G9a inhibition was mTOR-dependent and regulated by AMPK activation. G9a inhibition strongly inhibited the activation of mTOR pathway but activated the AMPK pathway. Inhibition of autophagy by 3-MA, Rheb overexpression (mTOR activator) or compound C (AMPK inhibitor) or activation of autophagy by AICAR (AMPK activator) significantly attenuated or enhanced the anti-proliferation effects of G9a inhibition, suggesting the anti-proliferation functions of autophagy induced by G9a inhibition. It is still unclear how autophagy induction contributes to proliferation inhibition and further studies are needed to illustrate the molecular mechanisms governing the interplay between them.

Epigenetic modifications including DNA methylation, histone acetylation and methylation, and microRNAs are described as stable inherited phenomena resulting from changes in gene expression without alternation in DNA sequence. It has been regarded as hallmarks of the initiation of tumorigenesis and aberrant epigenetic modifications have been considered to be pivotal mechanisms in the regulation of cell phenotypes[42]. Unlike apoptosis and cell cycle, of which the epigenetic regulation has been deeply understood, very little is known about epigenetic regulated autophagy. With the deeper exploration of tumorigenesis, more and more emphasis has been laid on epigenetic control of autophagy. Tumor suppressor genes (such as p53 and PTEN) have been discovered to be epigenetically silenced in cancers to modulate autophagy to influence tumor growth, malignant and resistance to anticancer therapies[43,44]. In addition, some molecular targets like BCL2 are revealed to be epigenetically regulated to affect signal transduction pathways to induce or inhibit autophagy[45]. Moreover, some key autophagy related genes (such as LC3A, LC3B, ATG3, ATG5 and ATG7), which regulate autophagy directly, are demonstrated to be silenced or up-regulated by epigenetic mechanism like DNA methylation, histone acetylation and methylation, or microRNAs[46].

G9a is discovered to be an epigenetic suppressor of autophagy and G9a inhibition genetically or by its inhibitor (such as BIX-01294) intensively stimulates autophagy. However, the underlying mechanism seems quite complicate and also cell type dependent. In HeLa and SU86.86 cells, G9a inhibits autophagy by directly epigenetically silencing autophagy regulated genes LC3B, WIPI1 and DOR[47]. While ATG3, ATG5, ATG7 and ATG12 are upregulated to induce autophagy in neuro-blastoma cells when G9a is inhibited[32]. In MCF-7, SKBr3, and HCT116 cells, G9a inhibition induces autophagy by increasing intracellular reactive oxygen species production. Moreover, Serine-Glycine Synthesis Pathway is involved in the epigenetic regulation of autophagy by G9a inhibition in SHEP1 and U2OS cells[14]. In addition, G9a inhibition induces autophagy in head and neck squamous cell carcinoma via epigenetically increasing dual specificity phosphatase-4 (DUSP4) to inactivate ERK[12]. However, seldom light has been shed on the epigenetic control role of G9a on AMPK pathway as it is activated by G9a inhibition in TCC cells to induce autophagy. Recent studies reveals that Sirtuin 1 (SIRT1), a conserved mammalian NAD (+)-dependent protein deacetylase, has emerged as a key metabolic sensor to regulate AMPK[48,49]. Which gives implication that epigenetic modifications may participate in AMPK signaling. Since G9a is an epigenetic modification enzyme while its substrates include not only histone but also other proteins, and G9a inhibition activates AMPK, whether G9a can directly modify AMPK or its regulators and change its function is intriguing to explore and further researches are needed to elucidate it[50].

Taking together, our results indicate that G9a expresses highly in bladder TCC and G9a inhibition induces autophagy *via* AMPK/mTOR pathway, and the autophagy induced positively contribute to the inhibition of cell proliferation. These findings shed some light on the functional role of G9a in cell metabolism and suggest that G9a might be a therapeutic target in bladder TCC in the future

## Supporting Information

S1 Fig The effects of BIX-01294 on normal urologic cell lines and the doses and time dependent manners of autophagy induced by BIX-01294. **(A)** MTT assay was performed to assess the impact of different doses of BIX-01294 on normal cells viability after 48 h. Cells were treated with **(B)** different doses of BIX-01294 (0.25, 0.5, 0.75, 1 and 1.5 μM) for 24h or **(C)** treated with 1 μM of BIX-01294 for different time periods (0, 0.5, 2, 4, 8 and 12 h), H3K9me2 and autophagy marker LC-3 I/II was checked. Histone3 and β-actin were used as the loading control respectively. Blots are representative of three separate experiments.
(TIF)

S2 Fig The mRFP-EGFP-LC-3 fluorescence assay to illustrate autophagic flux induced by G9a inhibition and the transfection efficiency of Rheb. **(A)** Cells were transiently transfected with mRFP-EGFP-LC-3 plasmid for 24 h and then treated with designated treatments. The mRFP and EGFP images were merged and presented. Yellow and red puncta of each merged image were analyzed **(B)**. **(C)**RT-PCR to examine the transfection efficiency of Rheb in steady cloning of T24 and UMUC-3.
(TIF)

S3 Fig The effects of BIX-01294 on autophagy-related genes. After 24 h treatment with BIX-01294 (0.75, 1 and 1.5 μM), the expression of autophagy-related genes was checked by RT-PCR **(A)** and Western-Blot **(B)**. β2MG and β-actin were used as the control respectively. RT-PCR and blots are representative of three separate experiments.
(TIF)

S1 Table The target sequence used in shRNA. (DOCX)

S2 Table The primers used in RT-PCR. (DOCX)
