# Supplementary figures and images for: G9a Inhibition Induces Autophagic Cell Death via AMPK/mTOR Pathway in Bladder Transitional Cell Carcinoma
Source: PLoS One. 2015 Sep 23;10(9):e0138390. doi: 10.1371/journal.pone.0138390 (PMC4580411; doi:10.1371/journal.pone.0138390)

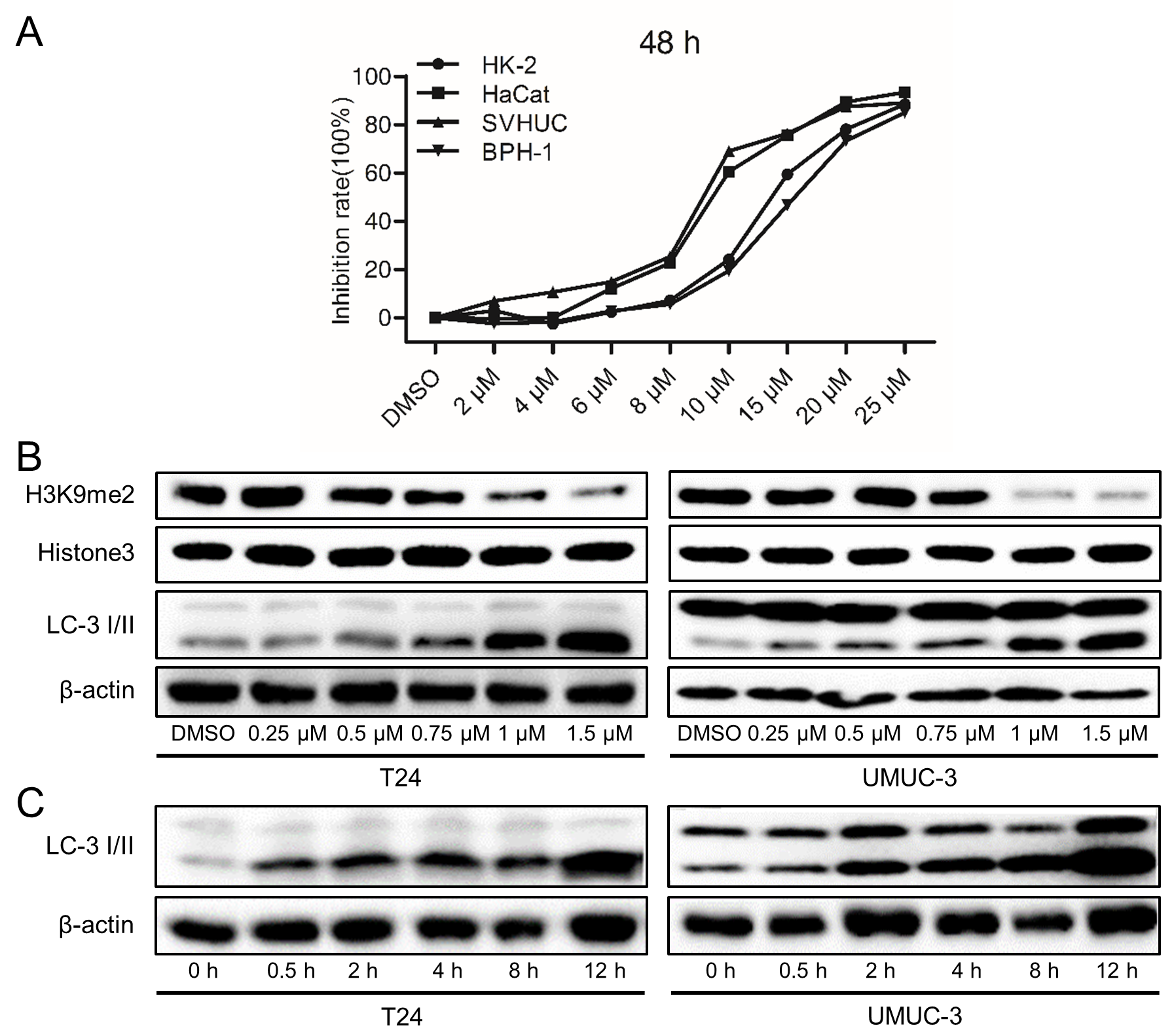

Supplement: S1 Fig — (A) MTT assay was performed to assess the impact of different doses of BIX-01294 on normal cells viability after 48 h. Cells were treated with (B) different doses of BIX-01294 (0.25, 0.5, 0.75, 1 and 1.5 μM) for 24h or (C) treated with 1 μM of BIX-01294 for different time periods (0, 0.5, 2, 4, 8 and 12 h), H3K9me2 and autophagy marker LC-3 I/II was checked. Histone3 and β-actin were used as the loading control respectively. Blots are representative of three separate experiments. (TIF) [file pone.0138390.s001.TIF]

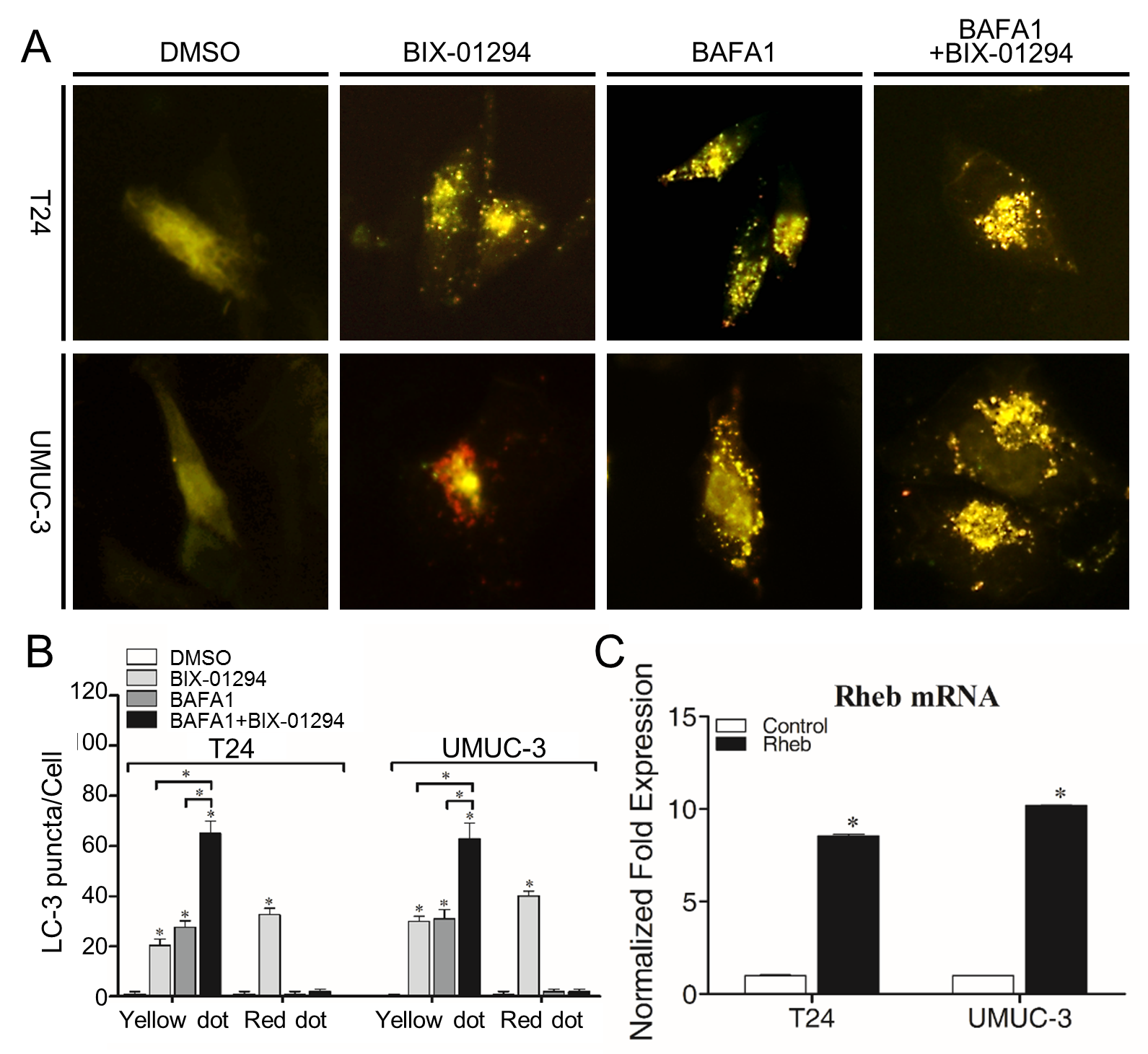

Supplement: S2 Fig — (A) Cells were transiently transfected with mRFP-EGFP-LC-3 plasmid for 24 h and then treated with designated treatments. The mRFP and EGFP images were merged and presented. Yellow and red puncta of each merged image were analyzed (B). (C)RT-PCR to examine the transfection efficiency of Rheb in steady cloning of T24 and UMUC-3. (TIF) [file pone.0138390.s002.TIF]

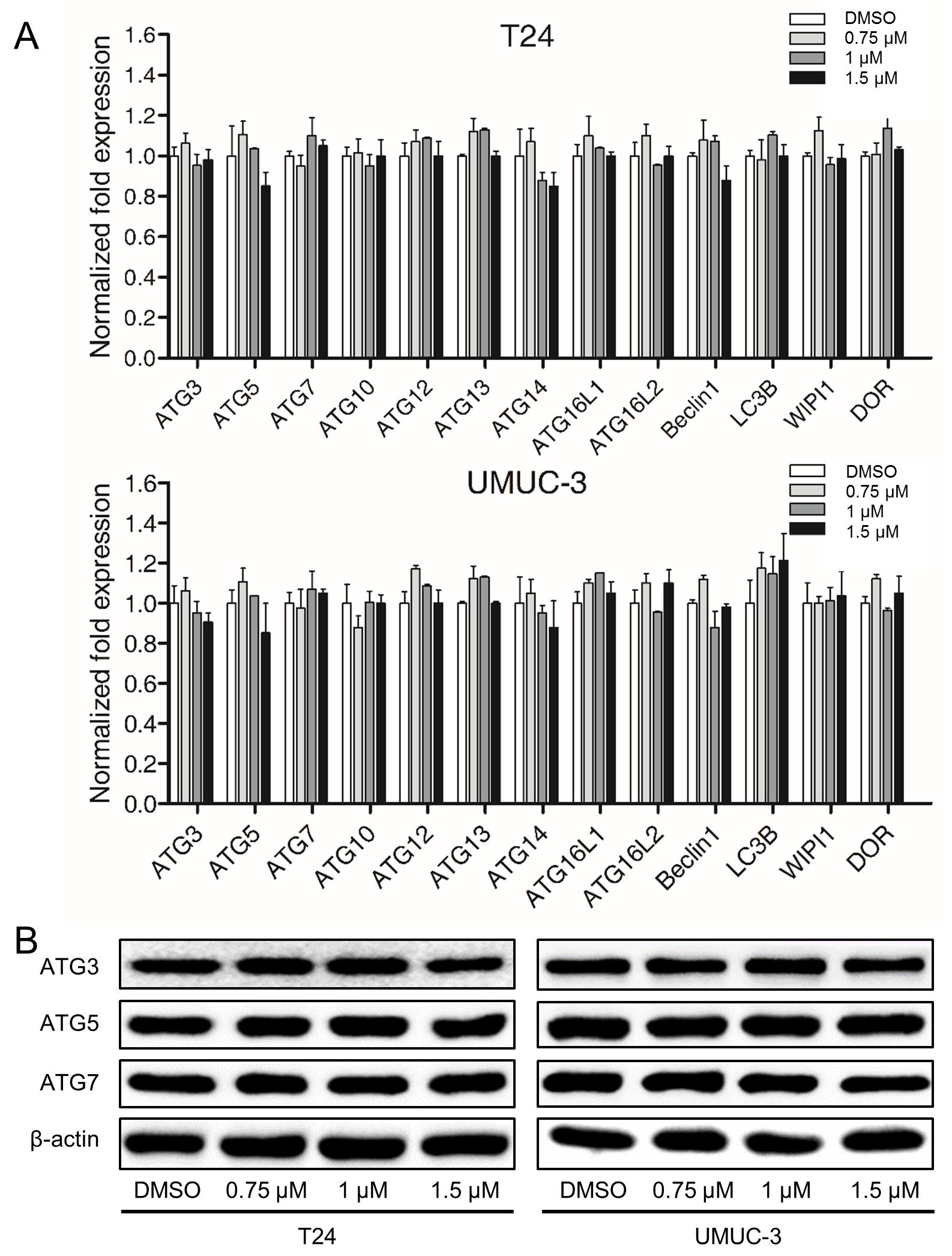

Supplement: S3 Fig — After 24 h treatment with BIX-01294 (0.75, 1 and 1.5 μM), the expression of autophagy-related genes was checked by RT-PCR (A) and Western-Blot (B). β2MG and β-actin were used as the control respectively. RT-PCR and blots are representative of three separate experiments. (TIF) [file pone.0138390.s003.TIF]
