# Supplementary material for: G9a Inhibition Induces Autophagic Cell Death via AMPK/mTOR Pathway in Bladder Transitional Cell Carcinoma
Source: PLoS One. 2015 Sep 23;10(9):e0138390. doi: 10.1371/journal.pone.0138390 (PMC4580411; doi:10.1371/journal.pone.0138390)
Supplement: S1 Table — (DOCX) [file pone.0138390.s004.docx]

| **shRNA name** | **Target sequence** |
| --- | --- |
| shG9a #1 | AGAGAGTTCATGGCTCTTT |
| shG9a #2 | TCCAGGAATTTAACAAGAT |
