# Supplementary material for: G9a Inhibition Induces Autophagic Cell Death via AMPK/mTOR Pathway in Bladder Transitional Cell Carcinoma
Source: PLoS One. 2015 Sep 23;10(9):e0138390. doi: 10.1371/journal.pone.0138390 (PMC4580411; doi:10.1371/journal.pone.0138390)
Supplement: S2 Table — (DOCX) [file pone.0138390.s005.docx]

| **Gene** | **Forward** | **Reverse** |
| --- | --- | --- |
| β2MG | GAGGCTATCCAGCGTACTCCA | CGGCAGGCATACTCATCTTTT |
| Rheb | TTGTGGACTCCTACGATCCAA | GGCTGTGTCTACAAGTTGAAGAT |
| LC3B | CCAGATCCCTGCACCATG | CTGCTTCTCACCCTTGTATCG |
| WIPI1 | ACGGTGCCAGGTTATTCTG | CGTTTTGCCCTTCTGATTTCC |
| DOR | CTTCTGTCCTCACTGTACCTTG | GAGAGGCGCTGGAACATG |
| Beclin1 | CCATGCAGGTGAGCTTCGT | GAATCTGCGAGAGACACCATC |
| ATG3 | GACCCCGGTCCTCAAGGAA | TGTAGCCCATTGCCATGTTGG |
| ATG5 | AAAGATGTGCTTCGAGATGTGT | CACTTTGTCAGTTACCAACGTCA |
| ATG7 | CAGTTTGCCCCTTTTAGTAGTGC | CCAGCCGATACTCGTTCAGC |
| ATG10 | AGACCATCAAAGGACTGTTCTGA | GGGTAGATGCTCCTAGATGTGAC |
| ATG12 | CTGCTGGCGACACCAAGAAA | CGTGTTCGCTCTACTGCCC |
| ATG13 | TTGCTATAACTAGGGTGACACCA | CCCAACACGAACTGTCTGGA |
| ATG14 | GCGCCAAATGCGTTCAGAG | AGTCGGCTTAACCTTTCCTTCT |
| ATG16L1 | AACGCTGTGCAGTTCAGTCC | AGCTGCTAAGAGGTAAGATCCA |
| ATG16L2 | TGGACAAGTTCTCAAAGAAGCTG | CCTCAGTGCGACCAGTGAT |
